# Long-term Formation of Aggressive Bony Lesions in Dogs with Mid-Diaphyseal Fractures Stabilized with Metallic Plates: Incidence in a Tertiary Referral Hospital Population

**DOI:** 10.3389/fvets.2017.00003

**Published:** 2017-01-31

**Authors:** Robert S. Gilley, Elizabeth Hiebert, Kemba Clapp, Lara Bartl-Wilson, Michael Nappier, Stephen Werre, Katherine Barnes

**Affiliations:** ^1^Department of Small Animal Clinical Sciences, Virginia-Maryland College of Veterinary Medicine, Blacksburg, VA, USA; ^2^Laboratory for Study Design and Statistical Analysis, Virginia-Maryland College of Veterinary Medicine, Blacksburg, VA, USA; ^3^Department of Veterinary Clinical Sciences, Louisiana State University School of Veterinary Medicine, Baton Rouge, LA, USA

**Keywords:** canine, fracture, implant, tumor, osteosarcoma, sarcoma, plate, bone

## Abstract

The incidence of complications secondary to fracture stabilization, particularly osteolytic lesions and bony tumor formation, has long been difficult to evaluate. The objective of this study was to describe the long-term incidence of aggressive bony changes developing in dogs with long bone diaphyseal fractures stabilized by metallic bone plates compared to a breed-, sex-, and age-matched control group. The medical records of a tertiary referral center were retrospectively reviewed for dogs that matched each respective criterion. Signalment, history, cause of death (if applicable), and aggressive bony changes at previous fracture sites were recorded. Ninety dogs met the criteria for inclusion in the fracture group and were matched with appropriate control dogs. Four of the dogs in the fracture group developed aggressive bony changes at the site of previous fracture repairs most consistent with osseous neoplasia. One lesion was confirmed with cytology as neoplastic. The population of dogs was mixed with regard to breed and body weight, but all dogs with aggressive bony lesions were male. Incidence of aggressive bony lesion formation in the fracture group was 4 (4.4%) and was 0 (0%) in the control group; three (75%) of the affected dogs in the fracture group included cerclage as a component of their primary fracture stabilizations. Incidence of aggressive bony lesions in the fracture group compared to the control group was determined to be statistically significant (*p* = 0.0455), as was the incidence of cerclage among dogs affected by aggressive bony lesions compared to the rest of the fracture group (*p* = 0.0499). Development of aggressive bony lesions is an uncommon complication of fracture fixation. Additional research is needed to further identify and elucidate the long-term effects of metallic implants in dogs.

## Introduction

Premalignant inflammatory changes and tumor formation in association with metallic hardware and fracture stabilization are a well-recognized, though uncommonly reported, occurrence in veterinary and human patients (1–3). Implant or fracture-associated tumors are neoplasms that develop in close proximity to a previous fracture or implant, develop after a considerable time delay between the inciting injury and implant placement, and lack other known predisposing factors in the vicinity including chronic infection or prior radiotherapy (2, 4–6). Previous reports of dogs afflicted with implant or fracture-associated tumors have identified primarily older patients, with an implant-to-tumor formation time range from 1 to 12 years (2, 4–6). Osteosarcoma is the most commonly identified tumor type (2, 6, 7). Implant- or fracture-associated osteosarcomas have distinctly different biological behavior compared to spontaneously forming osteosarcomas, as they are primarily reported to arise from the diaphyseal region of long bones rather than the metaphyses and are predominantly found in the femur and tibia rather than the radius or humerus (1, 2, 6, 8, 9).

Multiple mechanisms have been hypothesized to contribute to the formation of implant- or fracture-associated sarcomas, including sensitization of the patient to metallic implants, implant corrosion, genomic instability induced by prolonged metal exposure, delayed fracture unions, and healing of the fracture itself. Chronic tissue irritation secondary to low-grade infection has been shown to promote spontaneous tumorigenesis (10–20). Even with all of the potential risk factors, metallic implant-associated neoplasms in people are recognized as a rarity. The purpose of this study was to determine the incidence of aggressive bony lesions that may be implant-associated tumors in a population of dogs following open reduction and repair of long bone, diaphyseal fractures with metallic bone plates. Our hypothesis was that there would be a significantly higher incidence of aggressive bony lesions in dogs with fractures repaired with plates compared to a case-matched control group.

## Materials and Methods

The medical records available for review of dogs presenting with long bone fractures to a tertiary referral center from January 1, 2000 to April 1, 2014 were retrospectively evaluated. Cases were reviewed and dogs were selected based on method of fracture repair (open reduction and internal fixation using bone plates and screws) and fracture location (diaphyseal fractures). Exclusion criteria included non-diaphyseal fractures, amputation of the repaired limb less than 1 year after internal fixation, removal of the bone plate less than 1 year after internal fixation, pre-existing malignant neoplasia, fracture fixation-to-lesion interval of equal to or less than 1 year, or follow-up of less than 1 year. The time delay from implant placement to lesion formation was selected based on previous veterinary reports of implant-associated neoplasm formation as soon as 1 year after internal fracture stabilization (8, 20). Information obtained from medical records and phone interviews with the owners or referring veterinarians included signalment, history, comorbidities, fracture location, method of fracture fixation, and cause of death (if applicable). The medical records and radiographs of dogs identified with aggressive bony lesions more than a year postfixation at the prior fracture sites were reviewed by a board-certified veterinary radiologist. Radiographic findings consistent with aggressive disease include cortical destruction (which can include the presence of geographic, moth-eaten, or permeative lysis), presence of active periosteal proliferation (i.e., palisading, speculated, or amorphous new bone), and a longer zone of transition between normal and abnormal bone (21). Cytologic confirmation of aggressive bony lesions was recorded when available. All dogs that met the criteria to be included in the fracture group were compared to breed-, sex-, and age-matched controls from the same tertiary referral center with comparable follow-up information.

The primary outcome for statistical analysis of the fracture group was aggressive bony lesion formation. Incidence of aggressive bony lesions was compared between cases and age-, breed-, and sex-matched controls using McNemar’s chi-square test (because two cells in the 2 by 2 table had 0 counts, a small constant of 0.0001 was added to all four cells). Association between prognostic factors and development of aggressive bony lesions was assessed among dogs that underwent fracture repair (90 cases). Continuous prognostic factors were age at time of fracture repair and body weight, while categorical prognostic factors included gender, neuter status, breed, cerclage, non-cerclage metallic implants (not associated with securing the bone plate), non-metallic implants, bone, and side of body operated (right or left). Contingency tables were generated for the categorical factors. Association between aggressive bony lesion development and each of the continuous variables was assessed using the exact Wilcoxon rank-sum test. Associations between aggressive bony lesion development and each categorical factor were assessed with the Fisher’s exact test. Statistical significance was set to *p* < 0.05. All analyses were performed using SAS version 9.4 (Cary, NC, USA). Multivariable modeling was not successful due to the limited number of dogs with bony lesion formation. This limitation led to quasi-separation of data.

## Results

Of the 231 patients with long bone fractures reviewed, postoperative history and outcome or cause of death could be determined in 90 dogs. Median age and weight at the time of fracture were 22 months (range 1–160 months) and 12.3 kg (range 1.2–46.4 kg), respectively. The most frequently represented breeds were the mixed breed, Labrador retriever, German Shepherd Dog, and Chihuahua (*n* = 25, 6, 5, and 4). Forty-four of the dogs were male (20 intact, 24 castrated), and 46 were female (14 intact, 32 spayed). Median age at the date of last follow-up was 105 months (range 31–286 months). Each of the dogs in the fracture group was matched to a control dog of the same breed, sex, and comparable age (within 12 months) at the time of last follow-up. Forty-four of the dogs were male (11 intact, 33 castrated), and 46 were female (8 intact, 38 spayed). The median weight and age of the dogs in the control group at the time of last follow-up were 17.6 kg (range 0.8–68 kg) and 108 months (range 17–244 months), respectively.

Among the 90 cases in the fracture group with known outcome, 90 long bone, diaphyseal fractures repaired with metallic plates were identified. The radius was most frequently represented (42), followed by the femur (22), tibia (22), and humerus (4). Fifty-five fractures (61%) were repaired with bone plates alone, while 35 (39%) had additional implants, including cerclage (22, 24%), non-cerclage metallic implants (25, 28%), and non-metallic prosthetics (1, 1%).

Four of the patients in the fracture group with follow-up (4.4%) developed aggressive bony changes at previous fracture sites and ultimately were euthanized due to progression of clinical signs (Table 1). All of these patients presented with non-weight-bearing lameness and swelling with marked pain at the site of the bony lesions. Among the remaining 86 dogs, 63 were alive at inquiry; the other 23 dogs had either died or been euthanized for reasons unrelated to their fracture repairs. All dogs with suspected tumors were neutered males, with a median body weight of 25.1 kg. Median age of the dogs at the time of suspected tumor diagnosis was 113.5 months (range 91–177 months); median interval between fracture fixation and suspected tumor formation was 47 months (range 30–108 months). Three of the four dogs had additional implants in combination with the primary bone plate (three cerclage, one non-cerclage metallic implant). None of the patients in the control group developed clinically apparent lameness requiring radiographic evaluation or bone pain.

**Table 1:** **Signalment, age at time of fracture repair (AAF), age at lesion diagnosis (AAD), surgery-lesion interval (SLI), and bone affected of dogs that developed implant-associated lesions**.

Patient	Breed	Sex	Weight (kg)	AAF (months)	AAD (months)	SLI (months)	Bone
Case 1	German Shepherd Dog	MN	36.4	71	101	30	Humerus
Case 2	Standard Poodle	MN	20	18	126	108	Tibia
Case 3	Mixed breed	MN	30.1	146	177	31	Femur
Case 4	Miniature Schnauzer	MN	11.1	28	91	63	Femur

*MN, male neutered*.

**Figure 1:**
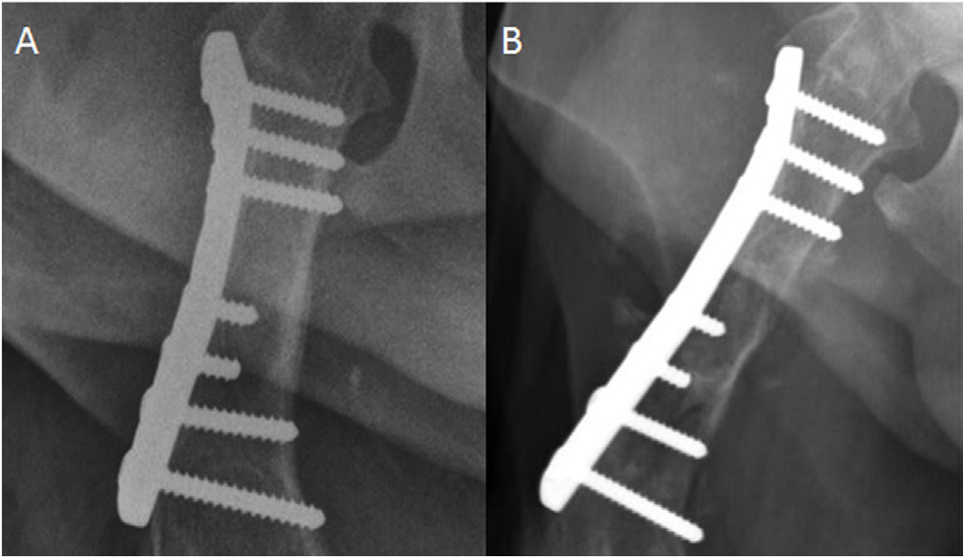
**Craniocaudal radiograph of the femur**. **(A)** This image was acquired immediately postfracture fixation. **(B)** This image was acquired 2 years later. There is now moth-eaten lysis of the proximal to mid diaphysis of the femur in addition to cortical thinning medially. Spiculated and amorphous periosteal proliferation is present medially and laterally, but is best observed lateral to the compression plate.

All dogs with bone lesions were confirmed to have radiographic signs consistent with aggressive disease. Radiographic findings in the affected dogs included moth-eaten and permeative osteolysis, primarily spiculated and amorphous periosteal proliferation, and circumferential soft tissue swelling all centered at the level of the implant (Figure 1). Aggressive changes were noted (*n* = 2) at the proximal aspect of the plate and the rest (*n* = 2) at the distal aspect of the plate. Based on the extended length of time between fracture fixation and the formation of monostotic aggressive bone lesions, implant-associated neoplasia was considered the top differential. Other possible differentials included naturally occurring primary osseous neoplasia, fungal osteomyelitis, and metastatic neoplasia. Of the four cases with osteolysis, one lesion was confirmed to be a sarcoma compatible with osteosarcoma by cytology. Cytologic confirmation was not available for three cases.

Variables analyzed for their effect on bony lesion formation within the fracture group included age at the time of fracture repair, body weight, neuter status, breed, cerclage, non-cerclage metallic implants, non-metallic implants, bone affected, and side of the body operated. Of those factors, only the presence of cerclage was found to be statistically significant. Table 2 lists *p*-values for all analyzed variables. When comparing the incidence of bony lesion formation in the fracture group to the incidence in the control group, the difference was found to be statistically significant (*p* = 0.0455).

**Table 2:** **Association between prognostic factors and bony lesion formation—fracture group**.

Prognostic factor	*p*-Value
Gender	0.0531
Neuter status	0.293
Breed	0.4308
Cerclage	0.0499^a^
Non-cerclage metallic implants	1
Non-metallic prosthetics	1
Bone	0.0775
Side operated	0.6657
Body weight (kg)	0.0862
Age at fracture repair (months)	0.1426

*^a^Statistically significant*.

## Discussion

Due to the incidence of spontaneous osteosarcoma in dogs, much of the debate regarding fracture-associated tumors centers on whether such tumors are coincidental events. The results of this study indicate a greater than expected incidence (4.4%) of late aggressive bony lesion development in dogs that have undergone internal fixation of diaphyseal long bone fractures with bone plates. Based on the data of this study combined with recent reports from veterinary literature, the authors believe that the number of dogs developing late bony lesions at sites of previous fracture repair is significant and has the potential to represent a unique entity. A similar relationship has been reported for implant-associated neoplasia and may be applicable to the one case with a diagnosis of neoplasia in our study (2, 6).

The affected dogs of this report were four neutered males, and two of the dogs represented were greater than 30 kg. Male dogs were exclusively affected in this study, but it should be noted that in two recent studies of fracture-associated and implant-associated neoplasia either female dogs were overrepresented or the sexes were equally affected (2, 9). Due to the low number of affected dogs in this report any sex predispositions cannot be evaluated. Previous reports of both implant-associated neoplasia and spontaneous osteosarcoma have shown predispositions for large to giant breed dogs, which was supported by our findings (1, 2, 9). It is, however, interesting to note that the smallest dog represented weighed only 11.1 kg and was a miniature Schnauzer, a breed not generally considered to be at increased risk of spontaneously developing osseous neoplasia. In one study, 4 out of 10 dogs with previously documented medullary bone infarcts that developed spontaneous osteosarcomas were miniature Schnauzers (7). No bone infarcts were documented to have formed in the miniature Schnauzer of this report. Median time from fracture stabilization to diagnosis of disease was 47 months (1,410 days), with the earliest bony lesion formation noted at 30 months (900 days) and the latest lesion formation noted at 108 months (3,240 days). A definitive diagnosis of neoplasia was achieved in only one patient of this report. However, it is worth noting that the affected dogs in this study have a time interval to lesion diagnosis comparable to a recent study of fracture-associated sarcomas in dogs, which reported fracture-associated sarcoma development between 1,348 and 3,488 days (9). The most commonly affected site in the patients of this report was the femur, consistent with a previous report identifying 12/13 dogs affected with histologically confirmed fracture-associated neoplasia occurring in the femur (9). Osteolytic lesion formation in all dogs in this study was believed to originate at the diaphysis, also consistent with a previous report of implant-associated neoplasia (2). While cytologic confirmation of neoplasia was achieved in one case; the remaining cases had radiographic changes strongly suggestive of a neoplastic process. However, other pathologic processes cannot be ruled out, including fungal disease.

Another interesting discovery was that three of the four dogs with aggressive bony lesion formation had additional metallic internal fixation (cerclage). This variable was found to be statistically significant (*p* = 0.0499) and was an unexpected finding. Due to the low number of dogs identified with aggressive bony lesions, this finding should be interpreted with some caution. While chronic irritation, inflammation, or infection has not been directly linked with implant-associated neoplasia, other tumors have been reported to form in association with these conditions (11, 15, 16). Thus, a similar relationship may be responsible for the lesions seen in the present study. Considering this, the authors speculate that if cerclage wire is not tightened optimally, or loosens over time, it may create chronic irritation and inflammation from micromotion, or infection; this may have contributed to the formation of the lesions seen. However, the reasons for the significant effect of cerclage wire are unknown and the occurrence of the lesions when cerclage was used may simply be coincidental.

Many hypotheses exist regarding the pathogenesis of implant-associated sarcomas. It has been proposed that implant corrosion and wear debris contribute a direct mutagenic effect to surrounding tissues (22). In support of this hypothesis, multiple studies have demonstrated that metallic implants are not biologically inert or categorically benign. Several metals, such as beryllium, cadmium, chromium, cobalt, and nickel, have been recognized as carcinogenic and metallic corrosion products have been identified locally in both animals and people (13, 17, 18, 23–25). Chromosomal aberrations have been found in human cells exposed to metallic corrosion and wear debris, and corrosion debris has also been found in veterinary and human patients with implant-associated tumors (13, 18, 22, 24, 26). In addition, laboratory animals exposed to metals used in metallic implants have developed tumors associated with the implant sites (8, 27).

While the lesion in one case was documented as neoplastic, it is not known if the plates used for the patients in this study contributed to the formation of the lesions seen. The plates used were all of 316L stainless steel and conformed to ASTM F138, Standard for Stainless Steel Bar and Wire for Surgical Implants (28). This is the present standard and was in effect during manufacture of all the plates used in this study. Thus, no differences in the composition of plates over the study period or the time surgery was performed can account for the occurrence of lesions observed. The use of dissimilar metals at the same fracture site, either from a lack of plate homogeneity or metallic composition differences between the plate and other implants, has been reported to increase the rate of plate corrosion (14, 23). However, a definitive mechanism for this increase was not clearly identified in these reports. As with the plates in this study, the cerclage wire used also was 316L stainless steel and conformed to ASTM F138 as a standard. While the metal for cerclage wire is worked to provide different mechanical properties, the specifications for the 316L stainless steel are the same. Considering this, different metals were not used and thus cannot account for the formation of the lesions observed.

Another hypothesis of the pathogenesis of implant-associated tumor formation focuses on the effects of chronic inflammation. Any factor that stimulates chronic inflammation may predispose patients to form tumors (11, 15, 16). Tissue sensitization has been documented in response to metallic implants in people (10, 12, 19). This hypersensitivity may be responsible for long-term implant loosening and chronic osteomyelitis (19). Chronic osteomyelitis, either due to implant–tissue interactions or from other underlying disease, has been associated with tumor formation in canine and human patients. However, while recognized as a contributor to tumorigenesis, definitive proof that implant-associated malignances are affected by long-term inflammation at the surgical site is not available (3, 16). Repetitive, low-grade trauma, bone infarcts, and the healing processes associated with sustaining a fracture, have also been considered as potential promoters of malignancy (7, 20, 29). Despite these findings, direct causal relationships are difficult to prove.

Limitations of this study include its retrospective design, a lack of tissue-based diagnosis for three dogs with radiographic evidence of aggressive bony disease at the site of previous fracture fixation that would support or confirm a neoplastic diagnosis, and the small sample population. Owner preference determined whether a definitive diagnosis was found for each case; for some owners, the cost and potential risks of bone biopsy or limb amputation were of enough concern that they chose not pursue a definitive diagnosis (30). In addition, all the dogs in this study that had bony lesions were non-weight-bearing lame and markedly painful at the lesion site and this likely contributed to the decision for euthanasia by the owners. Although cytology was not available for most cases, implant-associated neoplasia was considered most likely because the bony changes were centered at the level of previous surgical stabilization and took at least 2 years to form. However, because several cases lacked a tissue-based diagnosis, naturally occurring neoplasia and fungal disease remain potential differentials for the aggressive lesions identified. Additionally, it should be noted that many cases were excluded from the study group due to lack of adequate follow-up. This may have influenced the incidence of aggressive bony lesions identified in this report. A recent study of fracture-associated neoplasms reported an incidence of 0.0008%, which is considerably lower than the incidence of dogs that developed aggressive bony lesions in this report (4.4%) (9). While aggressive bony lesions cannot be diagnosed as neoplasia without cytologic or histopathologic confirmation, the authors speculate that radiographic appearance of these lesions alone is sufficient for many general practitioners to make practical decisions regarding the care of affected dogs. Furthermore, since both recent studies evaluating fracture and implant-associated neoplasms did not have any follow-up with general practitioners or owners, and exclusively used an electronic records system to identify cases, additional dogs affected with neoplasia could have been missed (2, 9).

Our hypothesis that there would be a significantly higher incidence of aggressive bony lesions in dogs with fractures repaired with plates compared to a case-matched control group was supported in the present study. Thus, the authors consider the findings of this study to be highly suggestive of a causal relationship between fractures stabilized with metallic implants and the development of aggressive bony lesions. Open reduction with internal stabilization is considered the treatment of choice for many fractures encountered in veterinary medicine (31). The authors feel strongly that the benefits of implants greatly outweigh the risk of late aggressive bony lesion development. This risk, however, may be increased in male dogs or animals that have cerclage wire used, based on the results of this study. Despite this finding, the increase in risk found for either parameter also may have been coincidental. Interestingly, the only case with a cytologic diagnosis of neoplasia did not have cerclage wire used; thus no direct relationship with cerclage wire and neoplasia can be made. Other than standard indications (implant failure, infection, irritation, athletes, and young animals), we do not advocate routine retrieval of implants due to the associated risks of general anesthesia, postoperative morbidity, and refracture of the initial repair site. A prospective study for the lifetime of a large population of dogs may provide the most accurate incidence of aggressive bony lesion development after fracture repair with plates. A prospective study may also aid in identifying additional risk factors for lesion formation, such as breed, nature of initial injury, implant composition, and fracture-associated complications.

## Ethics Statement

This study is a retrospective examination of patient clinical case records only. Thus, this study had no requirement for approval by the Virginia Tech Institutional Animal Care and Use Committee.

## Author Contributions

All authors (RG, EH, KC, LB-W, MN, SW, and KB) have provided the following contributions to the manuscript: (1) substantial contributions to the conception or design of the work; or the acquisition, analysis, or interpretation of data for the work; (2) drafting the work or revising it critically for important intellectual content; (3) final approval of the version to be published; and (4) agreement to be accountable for all aspects of the work in ensuring that questions related to the accuracy or integrity of any part of the work are appropriately investigated and resolved.

## Conflict of Interest Statement

This research was conducted in the absence of any commercial or financial relationships that could be construed as a potential conflict of interest.
